# Kali Carbonicum

**Published:** 1887-07

**Authors:** A. McNeil

**Affiliations:** San Francisco, Cal.


### KALI CARBONICUM.

(Carbonate of Potash.)

A LECTURE BY PROFESSOR A. MCNEIL, M. D., SAN FRANCISCO, CAL.

This antipsoric has been undeservedly neglected. The pivotal symptom around which all others revolve is the *stitching pains.* The patient will perhaps describe them as cutting, shooting, or stitching. Nearly all the pains which this drug cures, wherever they may be located, are of this kind. The patient may complain of headache, toothache, colic, angina pectoris, pains in the joints or viscera or elsewhere, but they are stitching. The drug whose pains have a striking resemblance in this regard is Bryonia. In fact, the pains are so much alike that many times we may be aided by remembering that Kali carb. is an antipsoric and consequently is more adapted to chronic diseases, while Bryonia, being a non-antipsoric, is for acute ones; and bearing this in mind, many times when Bryonia appears to be indicated and fails a closer examination of your patient will reveal psora in some of its many forms, and Kali carb. will be the remedy.

Kali carb. is adapted to fleshy and aged people, to complaints following parturition and abortion; in this Podophyllum resembles it, but the difference in cases to which each belongs is so striking that you can have no excuse for making a mistake except gross ignorance; in the bad effects of getting wet and standing in wet clothes, in this Dulcamara and Rhus tox. compete with it, Dulcamara especially in getting wet when cool and Rhus tox. when sweating.

After loss of fluids or vitality, particularly in the ænemic, in these patients you will of course always bear China in mind; a comparison of the symptoms will soon determine the choice. Then we have a class of patients who are œdematous and have a tendency to cachexy, as revealed by the bloating between the eyes and the brows.

There is a class of diseases in which this drug should always be in your mind, viz.: pulmonary consumption in its different stages; and here let me ask you not to make a fatal prognosis because you have diagnosed consumption. We have a few remedies that do in consumption what to our old school brother looks like the miraculous. In these cases you may safely drop from your mind all of the non-antipsorics, and among those that are left our remedy will often be the one. The stitching pains, particularly if in the lower part of the right lung is the seat of the disease, the time of aggravation of the cough from two to six o'clock A. M., will decide, and the flying from the mouth of hard, round, white masses when coughing and hawking. Here Kali carb., in the minimum dose and no repetition as long as improvement continues, will restore many an otherwise hopeless patient. We may also have with the cough a sensation of a lump rolling over and over and rising from the right abdomen up to the throat and then back again. There is also sputa of albuminous character mixed with small lumps of pus.

In diseases of women we will often have occasion to do good with this remedy. She feels very badly a week before menstruation. Her menses are too early, too profuse, and last too long, reminding us of Calcarea carb. She has some eructations every time she should menstruate; before her menses she has swelling of the pudendum; has violent itching of the whole body during the menses; with Carbo veg. there is itching between the shoulder blades; with Sarsaparilla itching on the forehead; she has swelling of the cheeks during the menses. Her menstrual discharge is pale, acrid, of a pungent, fetid odor, excoriates the parts with which it comes in contact (like Phosph. and Sulphur) and covers them with an eruption.

The characteristic stitching pains are in and about the uterus and its appendages. She has a tearing in the left labium extending through the abdomen to the chest. Murex purpurea has a pain extending from the right side of the uterus upward to the left side of the chest. She has soreness, gnawing, burning, and itching in the vulva and sore and pinching pains in the vagina during an embrace. Sepia also has painfulness during an embrace. During pregnancy this remedy is frequently indicated; in threatened abortion during the second month with stitching pains; when patient's back aches so badly while she is walking that she wishes to lie down at once and says she "feels as if she could lie in the street" at such times, to obtain rest and relief, cannot walk farther, and must sit down.

When patient is in labor the pain begins in the back, and instead of coming around in front like a regular pain it passes off down the buttocks or glutei muscles, or pains are sharp and cutting across lumbar region, arresting the contractions. This is the remedy in the sequellæ of abortion and confinement (also Podoph.), backache, night-sweats, dry cough, emaciation, or metrorhagia.

Children need Kali carb. when they are restless and anxious at night, cry much, and reach for things without taking anything; one may need to compare with Chamomilla and Staphisagria. The children are irritable when waking, scream, strike about them, and do not wish to be spoken to; just as the child who requires Lycopodium.

We will also find that other diseases of the thoracic organs than consumption will require Kali carb. In pleurisy, when Bryonia appears to be indicated, but, owing to a complication with psora, it fails to cure, this is the remedy.

When pleuritic effusion is not absorbed and is becoming purulent, as manifested by night-sweats, emaciation, and the stitching pains are frequent, Kali carb. is your last resort. Sulph., Lycop., and Phosph. may be indicated by the totality of the symptoms.

With all of these diseases there will be changes of mind and disposition. The Kali carb. patient is of a sad and weeping mood (reminding us of Caust. and Ignat.). She weeps from sad thoughts in the evening like the Sepia patient, while the Phosph. one weeps in the twilight. She is fearful when alone (Lycopodium and Stramonium), most in the evening in bed. She is fearful and anxious on account of her disease (like Agaricus and Lilium patients). She is irritable when awakening, so that she screams, strikes, and will not be spoken to (Lycopod.). She is absent-minded, so that she is at a loss what to do or what to say; makes attempts to speak, but is finally obliged to give it up. These mental symptoms make the morbid picture complete and reliable.

Patient has a feeling as if the bed were sinking under her (similar to Bryonia and Rhus tox.). Her hair is dry and brittle, while with Psorinum it is much like it, being lustreless and rough.

*He* has weakness of sight after an embrace (with Carbo veg. he has roaring in the ears). He has stitches in the middle of the eyes, and swelling like a bag between the eyebrows and lids, while the Apis patient has the swelling below the eyes.

His right ear is hot, and his left pale and cold. No other remedy has this singular symptom. His nose bleeds when washing his face (Arnica) and at nine A. M. He may have a dry coryza with total hoarseness and aphonia. He has a stitching pain in the pharynx, as if there was a fish-bone in it. Argent. nit., Hepar, Nitric acid have a similar symptom. The Kali carb. patient has many dyspeptic symptoms, he is annoyed by excessive flatulency, everything he eats or drinks appears to turn into gas (Argent. nit. and Iodine have the same). He belches putrid gas, like rotten eggs (Arnica and Psorin. the same). Has a sensation of emptiness, like Ign., Sepia, and others, but it is accompanied by eructations, and, similarly to Carbo veg., he belches much with relief.

The patient has nausea in every emotion and feels like fainting, and like the nausea of Bryonia and Coccul. it is relieved by lying down. She vomits, with a great faintness, and particularly when pregnant. She is worried by nausea coming on during a walk, while you remember the Pulsatilla patient is relieved when she is out-of-doors. Her stomach feels as if it were full of water. She is troubled with a sensation, as if a stick were extending from the throat to the left side of the abdomen, with a ball on each end of the stick.

The Kali carb. patient has bowel trouble, has constipation, with sensation when straining at stool as if the contents of the abdomen would be forced out of the vagina or rectum, and an unsuccessful desire for stool, with a sensation as if the rectum were too weak to expel it (Alumina and Hepar have the same). She feels very strangely and badly an hour before stool, and, on the other hand, she may have a diarrhœa only in the daytime (like Cocculus and Petrol.). She must be careful while passing flatus or an involuntary stool will pass. Aloes passes a hard stool while passing flatus, and Oleander and Verat. alb. diarrhœic ones. A diarrhœa at three or four o'clock A. M, and also one with the stitching pains so characteristic of this remedy.

Patient has hemorrhoids relieved by riding on horseback; this means relief by heavy pressure, such as sitting on something hard, which presses on the anus; of course, his piles are attended by stitching pains. Urine blackish (Lach.).

Our patient is frequently afflicted with heart trouble. He has a constriction in the region of the heart (like Cactus), when lying on right side, heart feels as if suspended to left ribs, and seems dragging them over to the right side. In angina pectoris always think of Kali carb.; the stitching pains in the region of the heart, aggravated by moving, breathing, and even talking, appear to require Bryonia, but the chronic nature of the disease excludes it, and Kali carb., with the same heart pains, is an antipsoric, and is very frequently the indicated remedy, and does good work not only during the paroxysm but to *cure* the case.

The Kali carb. patient has back pains, uterine, rheumatic, and neuralgic. He has pains in the back and legs after eating. Back aches as if broken and as if bruised (like Arnica) during rest, not during movement (Rhus tox.). Has weakness in the back and legs (like Cocculus). Is awakened from sleep, with stitching pains in the back, at three A. M. has to walk about to get relief.

Kali carb. has proven itself an important remedy in hip disease and white swelling of the knee. The stitching pains serve to indicate it. Patient has stinging pains in joints and inner parts (resembling Apis), fœtid sweat of the feet (like Silic. and Cuprum). She feels the pulsation of all the arteries (Iodine) even down to the tips of the toes; feeling of emptiness in the whole body, as if the body were hollow. Whole body feels heavy and broken down, so that it is only with the greatest effort that one can make any exertion.

These stitches, coming during perfect rest, are not dependent upon motion of any kind, and I have seen them cured with the aggravation from motion so characteristic of Bryonia.

Our patient has many nervous troubles. Her spasms seem to be relieved or pass off by frequent eructations, and the pains compel her to start and jerk; the least touch does the same. Her sleep is morbid, gets sleepy while eating, is troubled with nightmare as soon as she falls asleep (Cyclamen). She talks in her sleep and gets frightened; her complaints, like those of Lachesis, come after every sleep. She wakes in the morning about one or two o'clock, and cannot sleep from wakefulness, the Calc. carb. patient wakens after three, although the Kali patient may also waken at that hour. She wakes at this hour from cardiac anxiety and cannot go to sleep; when she lies on her right side she is anxious and has to sit up and belch.

She may have ulcers which, like those cured by Hepar, smell like old cheese, and she also has ulcers with hard edges which bleed at night.

Kali carb. is well followed by Phosph. and Nitric acid and is complementary to Carbo veg. Its resemblance to Bryonia I have already shown.

